# Subarachnoidal migration of intraocular silicone oil

**DOI:** 10.1007/s00701-017-3131-2

**Published:** 2017-03-03

**Authors:** Francisco J. Ascaso, Andrzej Grzybowski

**Affiliations:** 1 Department of Ophthalmology, Hospital Clínico Universitario Lozano Blesa, Av. San Juan Bosco 15, 50009 Zaragoza, Spain; 2 Aragon Health Research Institute (IIS Aragon), Zaragoza, Spain; 3 Department of Ophthalmology, Poznan City Hospital, Poznan, Poland; 4 Chair of Ophthalmology, University of Warmia and Mazury, Olsztyn, Poland

Dear Editor,

We have read with interest the article “Subarachnoidal migration of intraocular silicone oil” published by Cebula et al. [1]. We would like to congratulate the authors for the diagnosis and successful conservative management of two cases of such a rare complication.

We believe, however, that some discussion of this article is needed. Thus, we must clarify that what the authors describe as the posterior chamber of the eye really corresponds to the vitreous cavity. In fact, the posterior chamber, a narrow space located behind the iris but anterior to the lens, is a part of the ocular anterior segment. It is filled with aqueous humor and should not be confused with the vitreous chamber, which is part of the posterior segment of the eye and is occupied by vitreous humor.

We agree with the authors that the increased intraocular pressure or the presence of congenital optic nerve anatomical abnormalities might have caused intracranial migration of SiO from the vitreous space into the subarachnoidal space [2]. Nevertheless, they provide no information about either the aspect of the optic disc or the type and viscosity of the SiO employed in both cases. These clinical data regarding the presence of any of these predisposing factors in these patients might have played a crucial role in physical silicone migration [3].
